# Relationship between oxygen supply and cerebral blood flow assessed by transcranial Doppler and near – infrared spectroscopy in healthy subjects during breath – holding

**DOI:** 10.1186/1743-0003-3-16

**Published:** 2006-07-19

**Authors:** Filippo Molinari, William Liboni, Gianfranco Grippi, Emanuela Negri

**Affiliations:** 1 Biolab, Dipartimento di Elettronica, Politecnico di Torino, Torino, Italy; 2 S.C. Neurologia, Presidio Sanitario Gradenigo, Torino, Italy

## Abstract

**Background:**

Breath – holding (BH) is a suitable method for inducing cerebral vasomotor reactivity (VMR). The assessment of VMR is of clinical importance for the early detection of risk conditions and for the follow-up of disabled patients. Transcranial Doppler ultrasonography (TCD) is used to measure cerebral blood flow velocity (CBFV) during BH, whereas near-infrared spectroscopy (NIRS) measures the concentrations of the oxygenated (*O*_2_*Hb*) and reduced (*CO*_2_*Hb*) hemoglobin. The two techniques provide circulatory and functional-related parameters. The aim of the study is the analysis of the relationship between oxygen supply and CBFV as detected by TCD and NIRS in healthy subjects performing BH.

**Methods:**

20 healthy subjects (15 males and 5 females, age 33 ± 4.5 years) underwent TCD and NIRS examination during voluntary breath – holding. VMR was quantified by means of the breath-holding index (BHI). We evaluated the BHI based on mean CBFV, *O*_2_*Hb *and *CO*_2_*Hb *concentrations, relating the baseline to post-stimulus values. To quantify VMR we also computed the slope of the linear regression line of the concentration signals during BH. From the NIRS signals we also derived the bidimensional representation of VMR, plotting the instantaneous *O*_2_*Hb *concentration vs the *CO*_2_*Hb *concentration during the BH phase. Two subjects, a 30 years old current smoker female and a 63 years old male with a ischemic stroke event at the left middle cerebral artery, were tested as case studies.

**Results:**

The BHI for the CBFV was equal to 1.28 ± 0.71 %/s, the BHI for the *O*_2_*Hb *to 0.055 ± 0.037 *μ*mol/l/s and the BHI for *CO*_2_*Hb *to 0.0006 ± 0.0019 *μ*mol/l/s, the *O*_2_*Hb *slope was equal to 0.15 ± 0.09 *μ*mol/l/s and the *CO*_2_*Hb *slope to 0.09 ± 0.04 *μ*mol/l/s. There was a positive correlation between the CBFV and the *O*_2_*Hb *increments during BH (*r *= 0.865). The bidimensional VMR pattern shows common features among healthy subjects that are lost in the control studies.

**Conclusion:**

We show that healthy subjects present a common VMR pattern when counteracting cerebral blood flow perturbations induced by voluntary BH. The proposed methodology allows for the monitoring of changes in the VMR pattern, hence it could be used for assessing the efficacy of neurorehabilitation protocols.

## Background

Unlike the other organs, human brain needs a constant oxygen supply in order to maintain its functional and structural integrity. The local amount of oxygen stored in the brain tissues is small compared to the metabolic needs, hence a specific mechanism is necessary in order to ensure the correct oxygenation levels. This mechanism has to provide oxygen during both resting condition and focal cortical activity. The strict coupling existing between "activation", local oxygen consumption, and increased regional cerebral blood flow constitutes the basis of the so called BOLD effect (Blood Oxygenation Level Dependent) and, hence, of the functional magnetic resonance [1]. Thus, the assessment of cerebral hemodynamics is of paramount importance for determining the response of a subject to an external stimulus or for quantifying cortical activation.

Among the methods allowing a non – invasive and low – cost assessment of cerebral hemodynamics, transcranial Doppler ultrasonography (TCD) plays a fundamental role [2,3]. By means of TCD it is possible to measure the cerebral arteries blood flow velocity (CBFV) and, hence, analyze the variation of the CBF. However, the limited spatial resolution of this technique allows for the quantification of CBFV only in the macro – vessels (essentially the arteries constituting the Willis circle plus the middle cerebral arteries), whereas a cortical localized modification of blood velocity is impossible to track. Moreover, in about 25% of the patients, it is impossible to perform a TCD examination due to poor skull acoustic windows.

By means of near – infrared spectroscopy (NIRS) it is possible to continuously monitor the local concentrations of oxygenated (*O*_2_*Hb*) and reduced (*CO*_2_*Hb*) in the adult brain. TCD provides a direct measurement of circulatory parameters, whereas NIRS provides more functional and activation-dependent informations. Specifically, it has been demonstrated that NIRS can proficiently measure cerebrovascular reactivity [4].

In clinical practice, cerebral autoregulation is usually assessed during a *CO*_2 _reactivity test [5]. It is known that baroreceptors react to an increased partial pressure of *CO*_2 _by inducing vasodilatation in the resistance vessels; hence, the mean CBFV increases and the resistance of the vessels drops [6]. This mechanism is often indicated as vasomotor reactivity (VMR). *CO*_2 _reactivity can be induced by means of acetazolamide injection, by means of direct *CO*_2_inhalation (usually at the 5% – 7% concentration), or by means of simple breath – holding (BH).

In the last five years, a great variety of studies combining TCD and/or NIRS have been devoted to the assessment of VMR in subjects affected by acute and chronic pathologies: microangiopathy [7], migraine [8], carotid artery occlusion [9] and depression [10]. Recently, NIRS has been also used for the cerebral activity quantification during motion tasks [11]. From a rehabilitation point of view, NIRS proved successful in monitoring motor reorganization in hemiparetic stroke patients [12].

Traditionally, in response to a *CO*_2 _test, VMR is quantified by relating baseline values (these values can be the mean CBFV as well as the concentrations of *O*_2_*Hb *and *CO*_2_*Hb*) to post – stimulus values [9]; while the stimulus phase is not taken into consideration. Since VMR determines a continuous modification of such values during time, omitting the analysis of the stimulus phase may lead to uncertainties and poor comprehension of the VMR itself.

The aim of the study is the analysis of the relationship between oxygen supply and CBFV as detected by TCD and NIRS in healthy subjects performing BH. We studied a population consisting of 20 healthy volunteers and we showed the vasoreactivity patterns the subjects had during BH. We introduced a bidimensional representation of VMR based on the *O*_2_*Hb *and *CO*_2_*Hb *concentration changes that we consider useful to gain a better comprehension of VMR. Finally, we showed that this methodology could be used for assessing a subject's VMR condition, comparing the data of two case studies to those of the normal population.

## Methods

### Subjects

Currently, we enrolled in this study 20 (15 males and 5 females) healthy non-smokers volunteers (age, mean ± sd = 33 ± 4.5 years). Before being included in this study, all the subjects underwent clinical examinations intended to exclude cerebral, cardiac, and circulatory diseases. According to the rules of the local Hospital in which the tests were hold, the subjects were asked to sign an informed consent.

#### Case studies

We also tested several healthy current smokers subjects and some pathologic subjects. Due to the great variability of our sample population of smokers and pathologic subjects, we decided to present in this paper only two case reports which we found indicative of their category. The first subject was a healthy current smoker 30 years old female. She had been smoking for 12 years and she smoked an average of 15 cigarettes/day. The subject (indicated as subject A in the following) underwent the same clinical examinations of the normal controls and did not show any sign of cerebral, cardiac, and circulatory diseases. The second subject was a post-stroke, 63 years old, man. He had suffered from a ischemic stroke to the left middle cerebral artery (MCA) about 2 years before being enrolled in the study, when he was tested for the first time. He showed aphasia, motor impairment, and poor scores in fluency and verbal tests. After a year of drug therapy (antihypertensive and antiaggregating agents) and logopedic therapy, this subject was tested for the second time. He reported an improvement in motor control and reaching tasks, and increased his AAT (Aachener Aphasie Test) score from 52/60 to 56/60.

### Measurement protocol

We applied TCD and NIRS during baseline conditions and during *CO*_2 _reactivity. To trigger *CO*_2_reactivity, we chose the voluntary breath – holding technique. A major advantage of this choice is simplicity, since, to induce hypercapnia, there is no need for further devices (i.e. a capnograph with a breathing mask). This technique, however, is subject dependent: it is impossible, in experimental conditions, to establish a BH duration equal for all the subjects. To cope with this difficulty, we preliminary instructed the subjects on how to perform the BH and we let them test the procedure once before starting the recordings. In particular, we instructed the subjects to hold the breath after a normal breathing, in order to avoid an increase of the thoracic pressure, and we controlled they could hold the breath for a minimum time of 20 s. According to previously published experimental protocols, we instructed the subjects to end breath – holding when they felt comfortable [13].

The experimental protocol was the following:

• to derive baseline conditions, the subjects were allowed to rest for about 10 minutes in a dimmed and quiet room, laying comfortably in a supine position with eyes closed and breathing room air;

• when we observed stable signals (i.e. when the concentrations of *O*_2_*Hb *and *CO*_2_*Hb *and the CBFV did not show remarkable variations from their mean values), the subjects were instructed to perform a breath – holding after a normal inspiration;

• at the end of the apnea, the subjects were asked to rest for 5 minutes and we collected signals related to the post – stimulus conditions.

### TCD recordings

**Figure 1:**
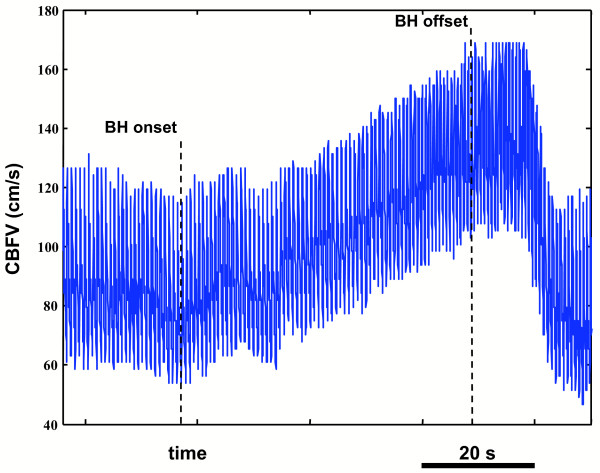
**CBFV modifications during BH of a healthy subject**. Time course of the CBFV during BH: the figure reports the entire Doppler spectra envelopes in function of time. The increase of CBFV is almost linear in function of the BH duration. After breath release, CBFV returns to baseline conditions quickly.

We recorded the CBFV in both the middle cerebral arteries simultaneously by means of a commercially available transcranial Doppler device (Multidop X4, DWL, Germany) equipped with 2 MHz probes. The transducers were positioned in order to insonate the MCAs in their Ml tract by the temporal bone windows. Probes positioning and the obtained Doppler sounds were confirmed on the basis of currently adopted clinical standards [14]. As an example, figure 1 depicts the modifications of the left MCA CBFV of a healthy subject performing BH. The figure reports the envelopes of the Doppler spectrum in function of time. It can be noticed how CBFV progressively and almost linearly increases while BH is maintained and then quickly recovers baseline conditions after breath release.

### NIRS recordings

Changes in the concentrations of *O*_2_*Hb *and *CO*_2_*Hb *were measured by means of a near – infrared spectroscopy device (NIRO 300, Hammamatsu Photonics, Australia). The emitting probe of the NIRS equipment was placed on the left frontal side of the subjects, 2 cm beside the midline and about 3 cm above the supraorbital ridge. We chose this positioning in order to avoid the sinuses and to place the probes on a poorly perfused and very thin skin layer. BH is supposed to induce a perturbation in cerebral cortex that is systemic and not regional or localized, hence the frontal lobe was a suitable location also for the absence of hairs. The receiving sensor was fixed laterally to the emitter at a distance of about 5 cm. According to previous studies and theoretical models already developed [15], we set a differential pathlength factor equal to 5.97. Previous works [15,16] demonstrated that with a source – detector distance equal to approximately 5 cm the NIRS equipment is capable of detecting effectively the chromophores concentration changes on the surface of the cerebral cortex.

**Figure 2:**
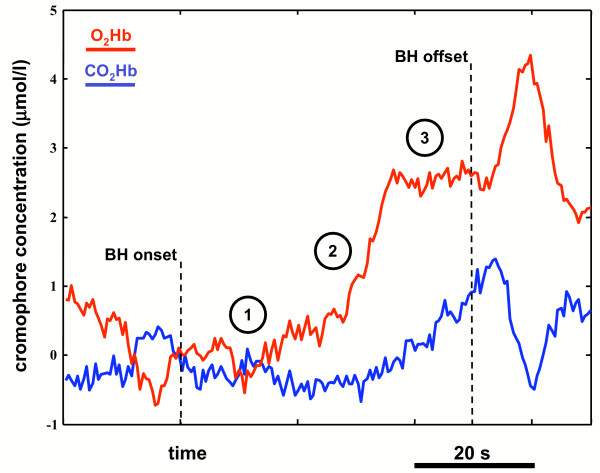
***O*_2_*Hb *and *CO*_2_*Hb *concentration changes during BH of a healthy subject**. Time course of the *O*_2_*Hb *(blue line) and *CO*_2_*Hb *(red line) concentration signals during BH. The graph is relative to a healthy subject. Values are scaled in order to set the initial (i.e., at the BH onset) concentration equal to zero. 1) Initial phase with concentration similar to the baseline values; 2) onset of vasoreactivity with strong *O*_2_*Hb *increase; 3) end of the vasoreactivity and plateau region for the *O*_2_*Hb *concentration, with increasing *CO*_2_*Hb *concentration.

Chromophores concentration changes were acquired continuously at a sampling rate equal to 2 Hz. To avoid bias from environmental light, a black cloth covered the NIRS probe. As an example, figure 2 reports the time course of the two types of hemoglobin during BH.

During the test, we also monitored the end-tidal *CO*_2 _and the mean arterial blood pressure by means of a specific monitor equipped with a capnographic module.

### Vasoreactivity quantification

According to previous studies [8], we used the breath – holding index (BHI) to quantify vascular reactivity. This index can be defined for any quantity related to the cerebral circulation, since it simply relates post – stimulus quantities to pre-stimulus quantities.

From the TCD data, we derived a BHI based on the mean blood flow velocity (MV). MV can approximately be defined as [17]:



where:

• PV is the peak systolic blood flow velocity;

• EDV is the end – diastolic blood flow velocity.

**Figure 3:**
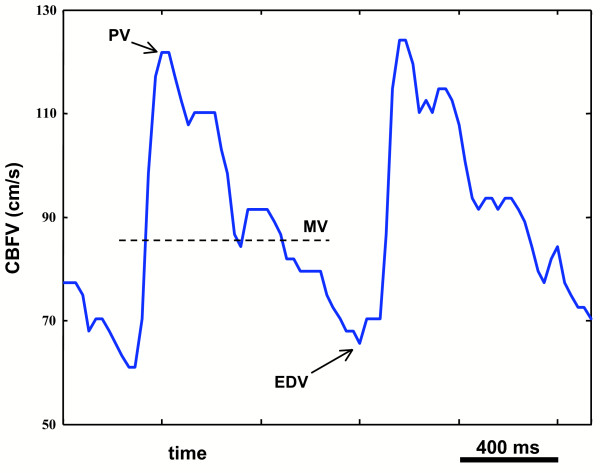
**Representation of the peak systolic, end diastolic and mean CBFVs**. Envelope of two waves of CBFV derived by a TCD scan of the left MCA of a healthy subject. The figure reports the indications of the peak systolic velocity value (PV), of the end diastolic value (EDV), and of the mean velocity value (MV) that are used for the calculation of BHI_*V *_and of the pulsatility index.

Figure 3 sketches the meaning of the PV, EDV, and MV in relation to the envelope of the CBFV during two cardiac cycles.

The BHI derived from the MV (which is indicated as BHI_*V *_in the following) was then defined according to the following expression:



where:

• *V*_*BASE *_represents the MV averaged on a 10s time window when in baseline conditions;

• *V*_*BH *_represents the MV averaged on a 10s time window after the offset of the apnea;

• *D*_*BH *_is the time duration of the BH.

This index is expressed in %/*s*.

From the TCD data, we also calculated the Gosling's pulsatility index (PI) of the MCA in baseline conditions and in correspondence of the maximum CBFV increase during the apnea. The PI is defined according to the following expression:



This parameter indicates how the ratio between the extreme velocities in the artery modifies as consequence of vasoreactivity and it is often used in VMR studies as a complement to the BHI [2]. To quantify VMR from the NIRS data, we estimated the chromophores concentration changes with respect to the BH duration [7]:



As in equation 2, *O*_2_*Hb*_*BASE *_is the oxygenated hemoglobin concentration in baseline conditions, averaged on the same 10s time window during which the *V*_*BASE *_is evaluated, and *O*_2_*Hb*_*BH *_is the average concentration after the release of the BH. We calculated the same index also for the *CO*_2_*Hb *().

These reactivity indexes are expressed in *μmol/l/s*.

**Figure 4:**
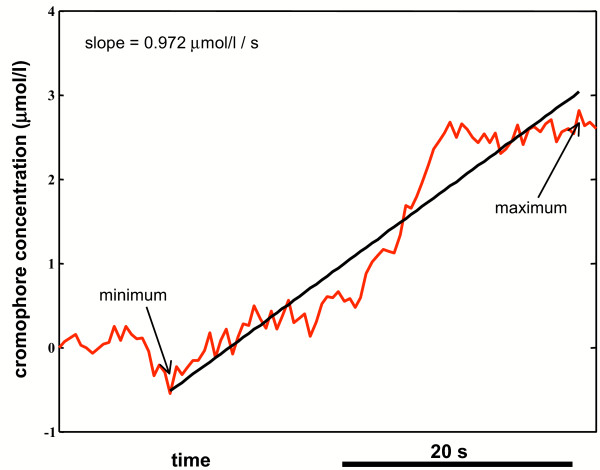
**Evaluation of the slope of the chromophore concentration changes**. Sketch of the slope computation for the *O*_2_*Hb *concentration signal of a healthy subject during BH: from the minimum and the maximum point of the concentration during BH, the angular coefficient of the linear regression line is computed. This slope is taken as index of VMR.

Beside the BHI, for each subject we also computed the slope of the *O*_2_*Hb *and *CO*_2_*Hb *concentration signals. Specifically, we evaluated the angular coefficient of the linear regression line traced from the minimum to the maximum concentration values on the chromophore concentrations time course during BH. Figure 4 depicts the regression line and the slope evaluation procedures for the *O*_2_*Hb *signal of a subject performing BH.

**Figure 5:**
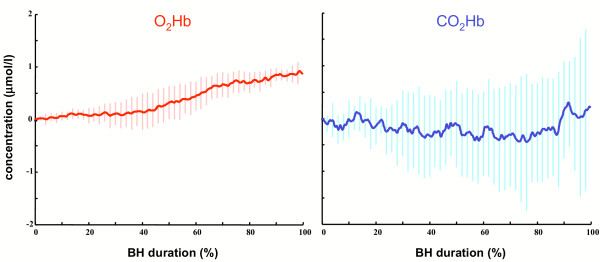
**Average *O*_2_*Hb *and *CO*_2_*Hb *concentration changes during BH**. *O*_2_*Hb *(left graph) and *CO*_2_*Hb *(right graph) concentrations during BH for the sample population. The superimposed vertical bars represent the standard error. The average graphs were obtained by normalizing the BH phase of each subject.

The mean variations of the *O*_2_*Hb *and of the *CO*_2_*Hb *were computed by first normalizing each BH duration and then averaging the chromophores concentrations on our sample population. The population averaged time course of the two NIRS signals are reported by figure 5.

### VMR bidimensional representation

**Figure 6:**
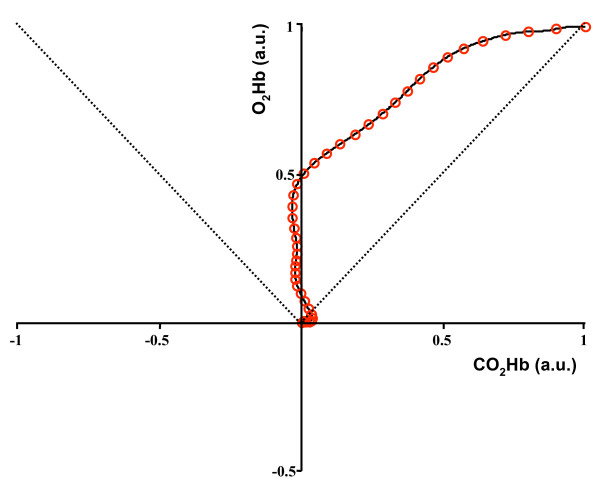
**Bidimensional VMR representation derived by NIRS signals**. Bidimensional VMR patterns as assessed by NIRS signals for the sample population. Each red circle represents the instantaneous concentration of *CO*_2_*Hb *(horizontal axis) and *O*_2_*Hb *(vertical axis). The concentration values are normalized with respect to their maximum value during the BH phase. The dotted lines depict the first and third quadrants bisectors. The reactivity pattern is always comprised into the region delimited by the two bisectors, evidencing a greater increase in the *O*_2_*Hb *level with respect to the *CO*_2_*Hb *concentration level.

To obtain the VMR bidimensional pattern during BH, we lowpass filtered the *O*_2_*Hb *and *CO*_2_*Hb *concentration signals (15 order Chebyshev digital filter, with ripple in the stop band, cutoff frequency equal to 50 mHz and at least 30 dB of discrimination) and set the initial concentrations equal to zero. The *O*_2_*Hb *and *CO*_2_*Hb *concentration signals were then normalized with respect to their maximum value during the BH phase. Then, in a bidimensional plane, for each time instant, we plotted the *O*_2_*Hb *vs the *CO*_2_*Hb *concentration. Lowpass filtering was introduced to obtain smooth profiles in the bidimensional representation; the zero setting of the initial conditions ensured that all the bidimensional patterns started form the graph origin, hence were direclty comparable. The resulting bidimensional plot are reported by figure 6.

## Results and discussion

### Carbon dioxide reactivity triggered by breath – holding

As already pointed out, the three major techniques adopted for triggering *CO*_2 _reactivity are: hypercapnia, acetazolamide injection, and breath – holding [5]. We decided to carry on this study using BH as reactivity trigger, since we planned to develop an experimental protocol that could be suitable for any subject, including patients suffering from cerebrovascular, neurological, and chronic diseases.

Breath – holding is obviously subject dependent; while this poses the problem of dealing with different BH durations, we believe this technique is suitable for assessing VMR as response to a sudden and abrupt change in the oxygenation levels, which is a major risk condition for cerebral autoregulation.

### VMR quantification

The population averaged BH duration was 41.7s ± 8.3s (95% confidence interval ranging from 38.1s to 45.4s). Table 1 reports the BHI_*V *_and the PI values derived from TCD measurements of the CBFV. The average increase in the CBFV was equal to 1.28 %/s ± 0.71 %/s, whereas the PI decrease from an initial average value equal to 0.86 to a post-apnea value of 0.66. These results are in line with previously reported studies concerning the use of TCD for the quantification of VMR [17]. From a methodological point of view, the neat decrement of the PI confirms that the experimental protocol was suitable for triggering vasomotor reactivity: during BH, the EDV increase was greater than the PV increase, hence PI diminished. Usually, the decrement of the PI is used to confirm the drop in the periferal vessel resistance, hence to ensure a correct onset of VMR.

**Table 1:** BHI and PI indexes derived from TCD signals. Population averaged values of the BHI and of the PIs derived from the TCD measurements. The first row depicts the percentage increment of the CBFV (BHI_*V*_), whereas the second and third rows depict the PI during baseline and after BH respectively. All the values are expressed as mean/sd.

	Mean/sd
*BHI*_*V *_(%/s)	1.28/0.71
PI baseline	0.86/0.13
PI BH	0.66/0.12

Table 2 summarizes the VMR indexes derived from the NIRS data. The first and second rows of Table 2 report the  and the  mean values for our testing population. The second column of the table reports the first species probability error in testing the corresponding value against zero (Student's *t *– *test*, *α *= 0.05), being zero the condition of no reactivity. We found that during voluntary BH, the subjects showed a significant increase in the *O*_2_*Hb *concentration level, whereas the variation of the *CO*_2_*Hb *was not statistically significant. The third and fourth rows of Table 2 report the average slopes of the *O*_2_*Hb *and of the *CO*_2_*Hb *concentration signals, computed as described in the materials section. Both the concentration signals were characterized by positive angular coefficients, but the slope of the *O*_2_*Hb *signal was greater than that of the *CO*_2_*Hb *(0.15/0.09 vs. 0.09/0.04, mean/sd).

**Table 2:** BHIs derived from NIRS signals. Population averaged values of the BHI and of the slope of the *O*_2_*Hb *and *CO*_2_*Hb *concentration signals derived from the NIRS data (all the values are expressed in *μ*mol/l/s). The first and the second rows report the BHIs derived from the concentration changes of oxygenated and reduced hemoglobin, the third and fourth rows report the slopes of the time course of the concentration signals during the BH phase (all the values are expressed as mean/sd). The second column reports the first species probability error of a Student's *t *– *test *to test the BHI and the slope values against zero (i.e. against no modification induced by the BH) with a confidence level equal to 95%.

	Mean/sd	P value
	0.055/0.037	4·10^-6^
	0.0006/0.0019	>0.05
	0.15/0.09	< 7·10^-7^
	0.09/0.04	< 5·10^-10^

We believe that the quantification of VMR by means of the BHIs derived by NIRS signals could be questioned. According to literature, vasomotor reactivity is quantified as the variation of a given physiological parameter as consequence of an external stimulus (usually a *CO*_2_increase). As a matter of fact, however, the above defined indices only depends on the baseline and on the post-BH conditions, but what happens during the BH phase is not taken into consideration.

Mean CBFV increases during *CO*_2 _reactivity tests as consequence of a pial arteries vasodilation, but then it remains almost constant for periods lasting several seconds [2]. Hence, the quantification of vasomotor reactivity based on pre-apnea and post-apnea values is appropriate. Conversely, as our experimental results clearly show, the local concentration of oxygenated hemoglobin measured by NIRS is a more rapidly evolving quantity, since it depends on the CBFV, on the perfusion pressure, on the degree of artery dilation and on the tissues oxygen extraction rate. Moreover, vasoreactivity is triggered by a *CO*_2_increase, but the quantification of VMR itself is usually done by taking into account the increases in both oxygenated and reduced hemoglobin; this because VMR is a functional physiological process aiming at maintaining a proper chromophores concentration in brain tissues. Hence, we believe that for a proper interpretation and evaluation of the VMR during BH it is necessary to observe the reactivity pattern during the apnea phase. We propose to measure the slopes of the *O*_2_*Hb *and of the *CO*_2_*Hb *concentration signals and to use them for quantifying VMR during voluntary breath-holding. This quantity, in fact, is strictly related to the time course of the hemoglobin concentration signal. This index is also implicitly normalized with respect to the BH duration; this enables direct a comparison of the results among different subjects.

**Figure 7:**
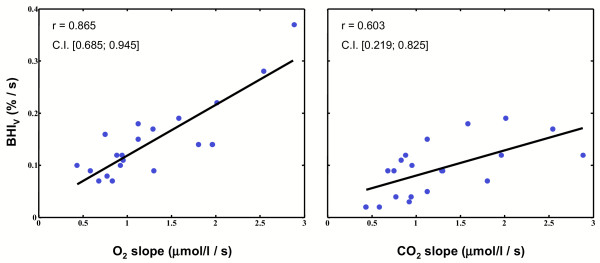
**Correlation between BHI_*V *_and slopes of the hemoglobin signals**. Scatter diagram of the BHI_*V *_and of the  (left graph) and  (right graph) values for the 20 subjects. The increment of the CBFV shows a good correlation with the increment of the *O*_2_*Hb*, which can be taken, in this experimental protocol, as an estimate of the increment of the cerebral blood volume.

Our results also revealed a good correlation between the BHI_*V *_and the slopes of the *O*_2_*Hb *and of the *CO*_2_*Hb *concentration signals: figure 7 reports the scatter diagrams of the BHI_*V *_and of the slopes (*O*_2_*Hb *on the left panel and *CO*_2_*Hb *on the right panel) for our sample population. The black line represents the linear regression of the data. The Pearson's correlation coefficients were found equal to 0.865 (BHI_*V *_vs slope of the *O*_2_*Hb *signal; *P *< 3·10^-7^, *α *= 0.05) and 0.603 (BHI_*V *_vs slope of the *CO*_2_*Hb *signal; *P *< 4·10^-3^, *α *= 0.05). The figure also depicts the 95% confidence intervals for the estimated correlation coefficients. The  and  did not show any correlation with BHI_*V*_. The variation of the *O*_2_*Hb *concentration, which is greater than that of *CO*_2_*Hb*, has a greater correlation with the increase in CBFV; this is not surprising since *O*_2_*Hb *concentration is predominant in the cerebral cortex. Approximating the increase of the regional cerebral blood volume with the *O*_2_*Hb *concentration increase, in healthy subjects performing our experimental protocol, an increase in CBFV is almost linearly correlated with the increase of the local cerebral blood volume.

### NIRS vasoreactivity patterns

As pointed out above, the BHI is a measure of VMR that relates the baseline to the post-stimulus values. Cerebral concentrations of *O*_2_*Hb *and *CO*_2_*Hb*, however, strongly vary during BH as consequence of vasodilation and of the local oxygen demand; thus, a more complete evaluation of VMR should be made by taking into account what happens during the BH phase.

Figure 2 reports an example of the changes occurring in the *O*_2_*Hb *(red line) and *CO*_2_*Hb *(blue line) concentrations during BH of a single healthy subject. Three main features can be observed on the time course of the two concentrations:

1. an initial phase, similar to the the baseline, in which the two chromophores concentrations do not significantly change;

2. the VMR phase, in which there is a strong increase of the *O*_2_*Hb *(and, hence, of the total hemoglobin, that roughly corresponds to the regional cerebral blood volume) while the *CO*_2_*Hb *is kept at a baseline level;

3. a plateau phase when the vasodilation has already reached its maximum, characterized by an almost constant level of *O*_2_*Hb *and a progressive increase of the *CO*_2_*Hb *level.

At the end of the BH, a recovery phase takes the concentration signals to baseline values. Despite the great variability affecting the NIRS signals, we found these common features in all the subjects we tested, provided that the BH duration was at least of 20 seconds. Figure 5 reports the population averaged *O*_2_*Hb *(left diagram) and *CO*_2_*Hb *(right diagram) concentration signals during BH. In order to make the signals comparable, we normalized the BH duration of each subject and set the initial concentrations (i.e., at the BH onset) equal to zero. The superimposed vertical bars represent the instantaneous standard error. Starting from 20% of the BH duration, the *O*_2_*Hb *signal depicts an increase in the variability that is due to the fact that, by that time, VMR had its onset. The linear increase of the *O*_2_*Hb *continues until 80% of the BH duration, then variability reduces and a region of plateau can be observed. Conversely, the *CO*_2_*Hb *shows a more variable behavior, but its average concentration remains at baseline values almost until the 90% the BH, when an increase, which cannot be further compensated, determines the end of the BH.

### Bidimensional VMR representation

Vasoreactivity is a physiological mechanism that ensures the correct brain oxygenation both in baseline conditions and dynamically in consequence of perturbations to the blood oxygenation level. Specifically, during hypoxaemia, the decrease of the arterial partial pressure of oxygen, and the consequent increase of the arterial partial pressure of carbon dioxide, triggers VMR. The mechanisms that determine the onset of vasoreactivity are still debated [18].

If TCD is useful to document the increased CBFV as a physiological response to an increased oxygen demand by the brain tissue and to estimate the drop of the pial arteries resistance, NIRS could be proficiently used to monitor VMR in relation to the local amount of oxygen consumption and extraction. To this purpose, we propose to observe the VMR pattern in a two-dimensional plane, where it is possible to monitor the instantaneous balancing of the two types of hemoglobin and to determine how autoregulation varies the concentration of the two chromophores.

**Figure 8:**
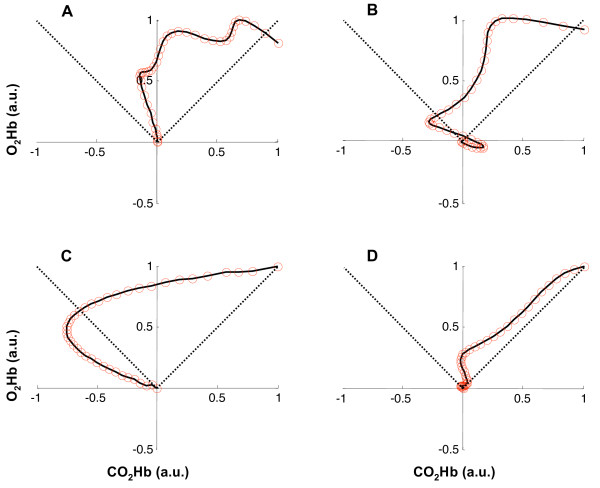
**Bidimensional VMR pattern for 4 healthy subjects**. Bidimensional reactivity pattern as derived by the NIRS signals for four healthy subjects. Each red circle represents the instantaneous concentration of *CO*_2_*Hb *(horizontal axis) and *O*_2_*Hb *(vertical axis). All the values are normalized with respect to the maximum. The dotted lines depict the first and third quadrants bisectors. All the graphs present characteristics of the VMR pattern of healthy subjects and are almost always comprises into the region delimited by the two bisectors. 15 subjects showed patterns similar to A and B, 4 subjects showed a pattern similar to graph C, whereas graph D is relative to the subject that showed the shorter plateau region.

Figure 6 reports the bidimensional BH patterns as assessed by means of the NIRS signals. The horizontal axis reports the instantaneous concentration of *CO*_2_*Hb *(normalized with respect to its maximum value during BH), whereas the vertical axis reports the *O*_2_*Hb *one (normalized with respect to its maximum value during BH). The dotted lines represent the first and third quadrant bisectors: when the VMR pattern is in the region comprised between the two bisectors, it means that the oxygenated hemoglobin concentration is increasing and, more specifically, it is increasing more than the reduced hemoglobin concentration. It is possible to notice that the VMR pattern is always comprised into this region. An initial increase in the *CO*_2_*Hb *concentration is rapidly compensated by a steep increase in the *O*_2_*Hb *concentration. Contemporarly, *CO*_2_*Hb *is kept at a concentration a little lower than the initial one. When the vasodilation has reached its maximum, there's a plateau region in which the *O*_2_*Hb *concentration remains almost constant, while the *CO*_2_*Hb *concentration starts increasing; afterwards, BH ends. This behavior was found for all the healthy subjects tested: figure 8 depicts the bidimensional VMR pattern for four different subjects. Even though the four patterns are different, there are common features that are characteristic of an intact autoregulation mechanism: i) after a very short initial phase, the VMR pattern is always comprised into the region delimited by the first and third quadrant bisectors; ii) *CO*_2_*Hb *is kept at baseline concentrations during the VMR phase, or, in some subjects, may decrease its concentration (graph C); iii) the final portion of the BH is characterized by a plateau region during which *O*_2_*Hb *is almost constant and *CO*_2_*Hb *tends to increase (a brief plateau region is observable in graph D, this pattern is relative to the subject that showed the minimum and shorter plateau phase).

A validation of these result is not straightforward: there are no studies, that we are aware of, that derived such bidimensional patterns from NIRS signals. However, the highly repeatable pattern we found in normal subjects suggests that cerebral autoregulation shows common features when counteracting the effects of BH. From a methodological point of view, we believe that the observation of the bidimensional pattern may be of help in interpreting more complex practical situations where autoregulation is impaired: in these conditions, a different balancing of the two chromophore concentrations could be expected. The following section reports two case studies, whose TCD and NIRS data are compared to our normative data.

### Case reports

#### Subject A – current smoker

**Figure 9:**
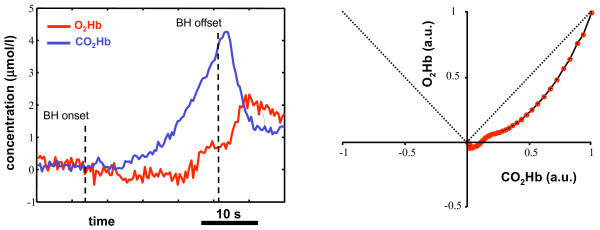
**NIRS signals and VMR pattern for subject A**. Time course of the *O*_2_*Hb *and *CO*_2_*Hb *concentration signals for subject A (healthy current smoker) during BH (left panel) and bidimensional VMR pattern (right panel). The signals reveal an uncompensated increase of the *CO*_2_*Hb *level, that determines a VMR pattern always out of the two bisectors region. Also, the onset of VMR is delayed and the VMR pattern never reaches a plateau condition.

This subject could voluntary hold the breath for 24 seconds, hence significantly less than the average of the normal controls. The first row of Table 3 summarizes the TCD and NIRS indexes for this subject. Similar to those of normal subjects were the BHI_*V *_(equal to 0.82 %/s) and the PIs before and after the BH (equal to 0.86 and 0.70 respectively). By means of the NIRS recordings, we computed a  similar to that of normal subjects (0.054 *μ*mol/l/s), but a greater  (0.051 *μ*mol/l/s). The slope of the *O*_2_*Hb *signal was equal to 0.132 *μ*mol/1/s and the slope of the *CO*_2_*Hb *was equal to 0.158 *μ*mol/1/s. These results are explained by the left panel of figure 9, which represents the time course of the two hemoglobin concentrations during BH. It can be noticed how *O*_2_*Hb *starts increasing only at the end of the BH phase, whereas *CO*_2_*Hb *rapidly increases during all the apnea and is never compensated. With respect to the average behavior of the normal population, this subjects depicts a delayed onset of VMR, a lack of increase in the *O*_2_*Hb *concentration, and an uncompensated increase of the *CO*_2_*Hb *concentration. Moreover, BH ends without reaching a plateau condition. The right panel of figure 9 shows the bidimensional VMR pattern derived by the NIRS data. It is evident that vasoreactivity is different from the pattern of normal subjects: the VMR pattern constantly moves in the 2D plane towards the increasing *CO*_2_*Hb *concentration direction and the increase in the *O*_2_*Hb *concentration is insufficient. As a consequence, the VMR pattern is never comprises between the two bisectors. Breath – holding, also, ends without reaching a plateau phase, hence it is impossible to state if this subject could compensate by reaching his maximum vasodilation. Several studies have already been devoted to the quantification of VMR in healthy current smokers (see [19,20] among others), even though results are not always in accordance each other: if some authors found a reduced cerebral blood volume during hypercapnia [21,22], other investigators did not find repeatable VMR patterns [23]. By means of our technique, we could document the delayed onset of VMR, the uncompensated *CO*_2_*Hb *concentration rise during BH, the VMR bidimensional pattern always out of the bisectors region, and the absence of a plateau region, that could stand for a chronic alteration of current smoking on the baroreceptor control [24].

**Table 3:** BHIs derived from TCD and NIRS signals for the case studies. Values of the BHI and of the slope of the *O*_2_*Hb *and *CO*_2_*Hb *concentration signals derived from the NIRS data for the two case studies. The first row reports the BH indicators for subject A, the second row reports the same indicators for the first test of subject B, and the third row reports the same indicators for the second test of subject B.

	BHI_*V *_(%/s)	PI baseline	PI BH	(*μ*mol/1/s)	(*μ*mol/1/s)	(*μ*mol/1/s)	(*μ*mol/1/s)
Subject A	0.82	0.86	0.70	0.054	0.051	0.132	0.158
Subject B – 1st test	0.05	0.61	0.64	0.0075	0.0005	0.015	0.0004
Subject B – 2nd test	0.9	0.63	0.60	0.046	-0.0048	0.026	0.046

#### Subject B – post-stroke subject

**Figure 10:**
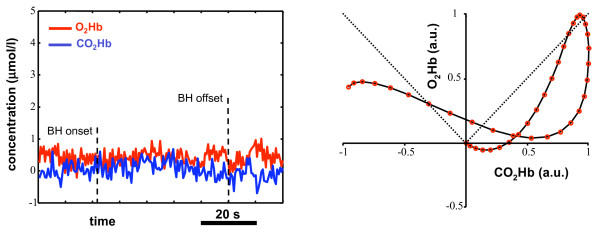
**NIRS signals and VMR pattern for subject B – 1st test**. Time course of the *O*_2_*Hb *and *CO*_2_*Hb *concentration signals for subject B (post-stroke subject) during BH (left panel) and bidimensional VMR pattern (right panel). Data are realtive to the first test, i.e. before the subject underwent therapy. The NIRS signals reveal the absence of vasoreactivity; the 2D pattern shows no functional organization.

During the first test, this subject could hold the breath for 47 seconds. Despite the good duration of BH, the second row of Table 3 reveals how VMR was strongly impaired: the BHI_*V *_was very small, and there was no drop of resistance in the peripheral vessels due to apnea (PI greater after BH than in baseline conditions). NIRS data confirmed this absence of VMR: , ,  and  were extremely low. Figure 10 (left panel) shows that there were no remarkable modifications in the *O*_2_*Hb *and *CO*_2_*Hb *concentrations during BH. The right panel of figure 10 depicts the bidimensional VMR pattern and confirms the absence of vasoreactivity: the hemoglobin concentrations change with no functionally significant coordination. Clinically, this subject suffered form an ischemic event to the left MCA, which determined a peripheral vasodilation and the onset of a compensatory circulation in the other branches of the Willis' circle. Hence, this subject was unable to react to a carbon dioxide increase since, to counteract the effects of stroke, its arteriolar bed was already in vasodilation conditions.

**Figure 11:**
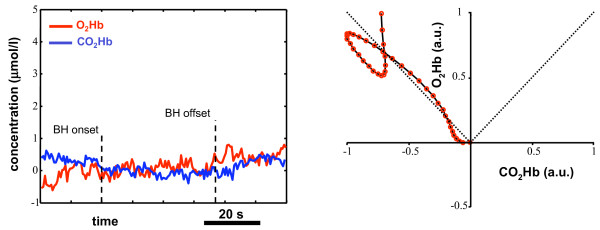
**NIRS signals and VMR pattern for subject B – 2nd test**. Time course of the *O*_2_*Hb *and *CO*_2_*Hb *concentration signals for subject B (post-stroke subject) during BH (left panel) and bidimensional VMR pattern (right panel). Data are realtive to the second test, i.e. after one year of drug and logopedic theraphy. The NIRS signals reveal an little increase in the *O*_2_*Hb *concentration that was not observable in previous examination; the 2D pattern shows that a functional response is present since *O*_2_*Hb *increases while *CO*_2_*Hb *is kept at low levels. This changes in the VMR data are in accordance with the clinical evaluation, which reported an improvement in motor and phasic scores.

After being treated with drugs and logopedic therapy for one year, the subject improved his motor and phasic performances. The results of the BH test reveal the effects of the therapy: the BHI_*V *_increases and the PI shows a drop during BH, meaning a little vasodilation is now present. Also, ,  and  increased, demonstrating that the subjects improved its reaction to the apnea. Figure 11 depicts the *O*_2_*Hb *and *CO*_2_*Hb *concentrations during BH (left panel) and the bidimensional VMR pattern (right panel) derived from the NIRS data collected after therapy. It can be noticed how the *O*_2_*Hb *presents greater variations during BH: these changes determine a bidimensional pattern that is, at least in a portion, comprised by the two bisectors. Moreover, VMR has now functionally sounding characteristics: *O*_2_*Hb *increases while *CO*_2_*Hb *is kept at low values.

Even though further studies are required, we believe this analysis methodology could be useful for monitoring and quantifying the effects of neurorehabilitation trials.

## Conclusion

In this paper we proposed a methodology for the assessment of VMR during voluntary BH. This methodology relates oxygen supply to cerebral blood flow by calculating BHIs based on TCD and NIRS data. We introduced a bidimensional representation of VMR during BH that we consider important to monitor the unbalancing between *O*_2_*Hb *and *CO*_2_*Hb *as consequence to a varied local oxygen demand.

On a population of 20 healthy subjects, we showed that the increment of the cerebral blood flow velocity in the middle cerebral artery is linearly correlated to the increment of the *O*_2_*Hb *when vasoreactivity is triggered by voluntary breath holding. Moreover, we provided normative BHI values on this sample population.

We observed that the vasoreactivity pattern of healthy subjects is characterized by common features that are not present if autoregulation is impaired: as an example we presented two case studies (a current smoker healthy subject and a post-stroke subject) and reported their BHIs and their bidimensional VMR patterns.

We believe these normative data could be useful when assessing vasoreactivity of subjects suffering both from chronic than acute pathologies with a direct impact on cerebral circulation.

From a methodological point of view, this joint analysis of TCD and NIRS signals could be used as a low-cost procedure for the bedside assessment of patients. Even though further studies are required in order to test the technique's performances, we consider this methodology as promising and we are planning protocols to monitor the effects of neurorehabilitation protocols in post-stroke patients.

## Competing interests

The author(s) declare that they have no competing interests.

## Authors' contributions

FM carried out the data analysis, participated in the experimental protocol design, and drafted the manuscript. WL designed the experimental protocol, participated in drafting the manuscript, and was responsible for the clinical evaluation of the subjects involved in the study. GG was responsible for the TCD data acquisition, participated in the TCD data analysis, and participated in the definition of the experimental protocol. EN was responsible for the NIRS data acquisition, participated in the NIRS data analysis, and participated in the definition of the experimental protocol. All authors read, commented, reviewed and approved the final manuscript.

## References

[B1] Ogawa S (1993). Functional brain mapping by blood oxygenation level-dependent contrast magnetic resonance imaging. A comparison of signal characteristics with a biophysical model. Biophys J.

[B2] Newell D, Aaslid R (1992). Transcranial Doppler.

[B3] Alexandrov A, Joseph M (2000). Transcranial Doppler: an overview of its clinical applications. The Internet J of Emergency and Intensive Care Medicine.

[B4] Smielewsky P, Kirkpatrick P, Minhas P, Pickard J, Czosnyka M (1995). Can Cerebrovascular Reactivity Be Measured With Near-Infrared Spectroscopy?. Stroke.

[B5] Provinciali L, Minciotti P, Ceravolo G, Sanguinetti C (1990). Investigation of cerebrovascular reactivity using transcranial Doppler sonography. Evaluation and comparison of different methods. Fund Neurol.

[B6] Piepgras A, Schmiedek P, Leisinger G, Haberl R, Kirsch C, Einhöupl K (1990). A simple test to assess cerebrovascular reserve capacity using transcranial Doppler sonography and acetazolamide. Stroke.

[B7] Terborg C, Felix G, Weiller C, Röther J (2000). Reduced Vasomotor Reactivity in Cerebral Microangiopathy. A Study With Near-Infrared Spectroscopy and Transcranial Doppler Sonography. Stroke.

[B8] Silvestrini M (2004). Basilar and middle cerebral arteries reactivity in patients with migraine. Headache.

[B9] Vernieri F (2004). Transcranial Doppler and Near-Infrared Spectroscopy Can Evaluate the Hemodynamic Effect of Carotid Artery Occlusion. Stroke.

[B10] Tiemeier H, Bakker S, Koudstaal P, MMB B (2002). Cerebral haemodynamics and depression in the elderly. J Neurol Neurosurg Psychiatry.

[B11] Kuboyama N, Nabetani T, Shibuya K, Machida K, Ogaki T (2004). The Effect of Maximal Finger Tapping on Cerebral Activation. J Physiol Anthropol Appl Human Sci.

[B12] Kato H, Izumiyama M, Koizumi H, Takahashi A, Itoyama Y (2002). Near-Infrared Spectroscopic Topography as a Tool to Monitor Motor Reorganization After Hemiparetic Stroke. A Comparison With Functional MRI. Stroke.

[B13] Safonova L, Michalos A, Wolf U, Wolf M, Hueber D, Choi J, Gupta R, Plzonetti C, Mantulin WEG (2004). Age-correlated changes in cerebral hemodynamics assessed by near-infrared Spectroscopy. Arch Gerontol Geriatrics.

[B14] Bartels E (1999). Color-Coded Duplex Ultrasonography of the Cerebral Vessels.

[B15] Okada E, Firbank M, Schweiger M, Arridge S, Cope M, Delpy D (1997). Theoretical and experimental investigation of near infrared light propagation in a model of the adult head. Appl Opt.

[B16] Firbank M, Okada E, DT D (1998). A theoretical study of the signal contribution of regions of the adult head to near – infrared spectroscopy studies of visual evoked potentials. Neuroimage.

[B17] Tegeler C, Babikian V, Gomez C (1996). Neurosonology.

[B18] Johnston A, Steiner L, Gupta A, Menon D (2003). Cerebral oxygen vasoreactivity and cerebral tissue oxygen reactivity. Br J Anaesth.

[B19] Terborg C, Bramer S, Weiller C, Röther J (2002). Short-term effect of cigarette smoking on CO2-induced vasomotor reactivity in man: A study with near-infrared spectroscopy and tanscranial Doppler sonography. J Neurol Sci.

[B20] Terborg C, Birkner T, Bärbel S, Witte O (2002). Acute effects of cigarette smoking on cerebral oxygenation and hemodynamics: A combined study with near-infrared spectroscopy and transcranial Doppler sonography. J Neurol Sci.

[B21] Kubota K, Yamaguci T, Abe Y, Fujiwara T, Hatazawa JTM (1983). Effects of smoking on regional cerebral blood flow in neurologically normal subjects. Stroke.

[B22] Rogers R, Meyer J, Shaw T, Mortel K, Hardenberg J, Zaid R (1983). Cigarette smoking decreases cerebral blood flow suggesting increased risk for stroke. JAMA.

[B23] Kimura K, Matsumoto M, Handa N, Hashikawa K, Moriwaki H (1993). Non-invasive assessment of acute effects of cigarette smoking on cerebral circulation. Yakubutsu Seishin Kodo.

[B24] Gerhardt U, Vorneweg P, Riedasch M, Hohage H (1999). Acute and persistant effects of smoking on the baroreceptor function. J Auton Pharmacol.

